# Evaluation of Minimum Recording Time and the Influence of Time in the Supine Position on Out-of-Center Sleep Tests

**DOI:** 10.1055/s-0043-1769495

**Published:** 2023-11-29

**Authors:** Raquel Chartuni Teixeira, Michel Burihan Cahali

**Affiliations:** 1 Department of Otolaryngology, Faculdade de Medicina, Universidade de São Paulo (FMUSP), São Paulo, SP, Brazil

**Keywords:** sleep apnea, obstructive, polysomnography, monitoring, ambulatory care

## Abstract

**Introduction** The prevalence of moderate to severe sleep-disordered breathing is of 17% among men aged between 50 and 70-years, and of 9% among women in the same age group. In Brazil, obstructive sleep apnea (OSA) is also highly prevalent, and it is associated with metabolic and cardiovascular impacts, excessive daytime sleepiness, and increasing risk of traffic accidents. Laboratory-based polysomnography is the gold standard test for OSA diagnosis. However, its complexity has led to the search for alternatives to simplify the diagnosis, such as the out-of-center sleep test (OCST).

**Objectives** To discusses the minimum OCST recording time and the potential effects of the supine position on this parameter.

**Data Synthesis** We conducted a search on the PubMed, Web of Science, Scopus, and Embase databases to identify relevant studies on OCST recording time and a possible association with body position. We used a combination of terms, including *Obstructive Sleep Apnea* and *Home Monitoring* OR *Home Care Services* OR *Portable Monitoring* AND *Supine* OR *Position* OR *Recording Time* OR *Positional Obstructive Sleep Apnea* . The references of the selected articles were also reviewed to find other relevant studies. Through our approach, eighteen articles were retrieved and included in the present study.

**Conclusion** Since OCSTs are conducted in an unattended environment, with potential signal loss during the night, it is crucial to determine the minimum recording time to validate the test and assess how the time spent in the supine position affects this parameter. After reviewing the literature, this topic remains to be clarified, and additional studies should focus on that matter.

## Introduction

Obstructive sleep apnea (OSA) involves airflow obstruction in the upper airway accompanied by desaturations or awakenings. 1 In addition to the classic manifestations of excessive sleepiness, non-restorative sleep, fatigue, and difficulty concentrating, OSA has been associated with notable cardiovascular and metabolic outcomes. 2 3 Moreover, a survey published in 2012 4 showed that 21% of traffic accidents involving deaths were caused by driver drowsiness. Therefore, the costs associated with not diagnosing or treating this pathology are significant.

In a study conducted by Peppard et al., 5 the prevalence of moderate to severe sleep-disordered breathing (apnea-hypopnea index [AHI], measured as events/hour, ≥ 15) was of 10% among 30 to 49-year-old men; of 17% among 50 to 70-year-old men; of 3% among 30 to 49-year-old women; and of 9% among 50 to 70-year-old women. In Brazil, the prevalence of OSA has increased, 6 which has been linked to the rise in cases of obesity. 7

According to the third edition of the International Classification of Sleep Disorders (ICSD-3), 8 OSA diagnosis requires polysomnography. In-laboratory polysomnography is the gold standard for OSA diagnosis, but it is considered a technically complex, expensive, and sometimes unavailable test. 9 Therefore, an interest in alternative diagnostic confirmation methods has increased.

Type-1 polysomnography (PSG1) assesses six electroencephalogram (EEG) channels, electrooculogram, chin and leg electromyogram, electrocardiogram, respiratory effort through piezoelectric strips on the chest and abdomen, respiratory flow (nasal cannula and thermistor), oxygen saturation and body position in a attended environment.

Level 3 portable monitors use two respiratory variables (effort and flow), blood oxygenation, cardiac variable (heart rate or eletrocardiogram). In 2007, the American Academy of Sleep Medicine (AASM) reviewed studies evaluating out-of-center sleep tests (OCSTs) for OSA diagnosis, including the PM3. 1 It was recommended the use of PM3 in patients with a high pretest osa probability and without relevant comorbidities.

In addition to the growing interest in simpler diagnostic methods, it remains unclear if PSG1 is the most appropriate exam for all patients. For example, the AHI was introduced in the last century and was important in defining OSA and differentiating it from other kinds of sleep-disordered breathing, such as obesity-related hypoventilation syndrome. However, in light of the current knowledge, the lack of homogeneity in the definition of hypopnea and the inability of studies to correlate the AHI severity ranges with negative outcomes have drawn attention to the analysis of new variables that could be considered in this heterogeneous pathology. 10 Indeed, oxygen saturation and other diagnostic technologies have acquired importance. 11 12 13 14

It is believed that using inexpensive and accessible technology in OCSTs could circumvent night-to-night variability, 15 enabling recording for consecutive nights. An unsupervised patient could monitor sleep for a very short time or have a signal loss during the night. That is why determining the minimum recording time is critical. The AASM recommends at least four hours of adequate recording for in-home polysomnography. However, there is not enough data to suggest that fewer than four hours of adequate recording should compromise the test's accuracy. 16

Furthermore, unsupervised patients could feel less reluctant to adopt non-supine positions in bed. 17 It is plausible that different sleep positions could significantly impact the diagnosis because many patients, especially those with a mild and moderate increase in the AHI, have an accentuation of obstructive respiratory events in the supine position. 18 It is estimated that more than 50% of patients with OSA present disrupted respiratory parameters in the supine position. In 1984, Cartwright was the first to randomly define positional apnea as one in which the supine AHI is at least twice as high as the non-supine AHI. 19 20 Notably, the medical literature cites several modified versions of Cartwright's classification. 18 19 20 Regardless of the classification used, it has already been demonstrated that a significant number of patients experience a marked effect of position on desaturations, cyclic variations on heart rate, loud snoring, and apneas and hypopneas, as demonstrated in 2017 by Ravesloot et al. 21

The high prevalence of position-dependent obstructive sleep apnea (POSA) points to the importance of evaluating the impact on the diagnosis of a device that, in principle, would enable the patient to spend less time in the supine position. Thus, in the present study, we conducted a literature review to identify studies on the ideal recording time in OCSTs and a possible influence on this parameter of the time spent in the supine position.

## Review of the Literature

We conducted a search on the PubMed, Web of Science, Scopus, and Embase databases to identify relevant studies on recording time and a possible association with sleep position. The searches combined the terms *Sleep Apnea, Obstructive* OR *Apnea, Obstructive Sleep* OR *Apneas, Obstructive Sleep* OR *Obstructive Sleep Apnea* OR *Obstructive Sleep Apnea Syndrome* OR *Obstructive Sleep Apneas* OR *Sleep Apnea Hypopnea Syndrome* OR *Sleep Apnea Syndrome, Obstructive* OR *Sleep Apneas, Obstructive* OR *Syndrome, Obstructive Sleep Apnea* OR *Syndrome, Sleep Apnea, Obstructive* OR *Syndrome, Upper Airway Resistance, Sleep Apnea* OR *Upper Airway Resistance Sleep Apnea Syndrome* AND *Home Monitoring* OR *Home Care Services* OR *Portable Monitoring* AND *Supine* OR *Position* OR *Recording Time* OR *Positional Obstructive Sleep Apnea* . The references of all selected studies were also reviewed to identify other relevant articles.

**Fig. 1:**
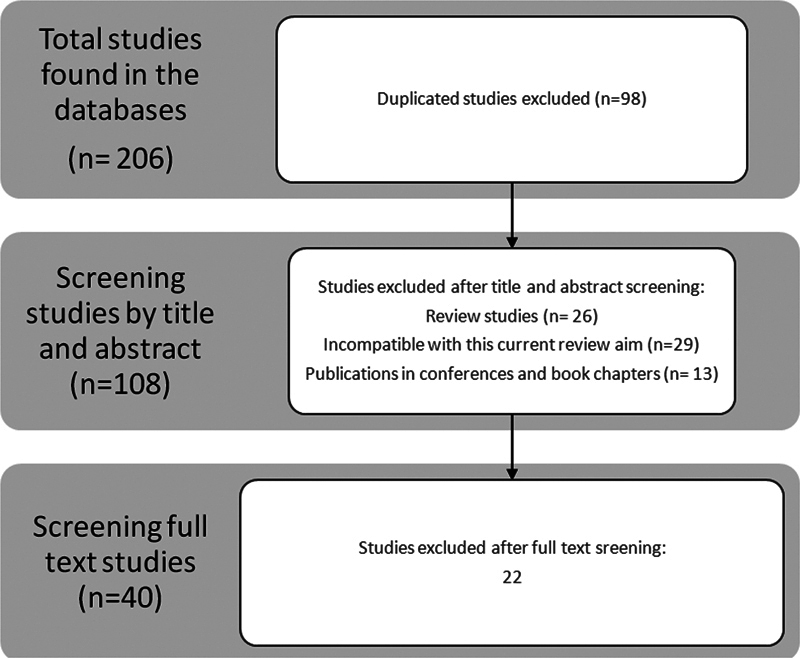
Selection o articles for the systematic review.

Prospective observational studies, clinical trials, and retrospective studies in patients with clinical suspicion of OSA were included. Narrative or systematic reviews were excluded. The search covered the past 25 years and was limited to individuals over 18 years of age who participated in PM3 studies. The studies were evaluated initially by two investigators who reviewed titles and abstracts. Studies that did not monitor flow, oximetry, and respiratory effort were excluded ( Fig. 1 ).

The data collected included: title; author; country; year of publication; the name of the publishing journal; keywords; study design; sample size; recording time; AHI; AHI in the supine position (AHIsup); total time in the supine position; sample size evaluated; and whether there was a comparison with PSG1.

The initial search in the databases yielded 206 articles, and we excluded 98 duplicate articles. Of the 108 remaining articles, 68 were excluded after an analysis of the titles and abstracts ( Fig. 1 ). After reading the full texts, 18 articles were ultimately selected ( Table 1 ).

**Table 1:** Studies selected after full text screening

Author	Study type	Number of Patients	Does it compare with PSG1?	Does it show the recording time (in minutes)?	Does it show the time (in minutes) spent in supine position?	Results
**Mello et al.** 17	**Cohort study**	**478 patients**	**Yes**	**Yes: 398 (355–434) for PSG1 and 423 (360–467) for PM3**	**Yes: 41.0 (16.4–66.7) for PSG1 and 33.9 (15.8–51.1) for PM3**	**PSG1 may increase time spent in the supine position and overestimate OSA severity**
**Wittine et al.** 22	**Cohort study**	**129 patients**	**No**	**Yes: 475 (446–479)**	**No**	**At least 300 minutes of recording are required for significant test accuracy**
**Cheliout- Heraut et al.** 24	**Clinical trial**	**90 patients suspected of OSA**	**Yes**	**seven recordings were excluded due to a total sleep time < 5 hours**	**No**	**Automatic event detection based on mandibular movement has important potential**
**Yin et al.** 27	**Clinical trial**	**44 patients**	**Yes**	**436,3±87,7 FOR pm3, 426,3 ±71,2 FOR PSG1**	**Yes: 62.7 ± 26.4% for PSG1 and 51.3 ± 30.0% for PM3**	**At least 390 minutes required in the PM3, longer supine time in the PSG1.**
**Planès et al.**	**Clinical trial**	**50 patients**	**Yes**	**Yes: 346 ± 62 (105–438)**	**No**	**PM3 is suitable for coronary artery disease patients**
**Driver et al.** 25	**Clinical trial**	**73 patients suspected of OSA**	**No**	**239 (62-409) for PSG1 and 379 (58-467) for Pm3**	**No**	**Low registration time reduced the agreement between PSG1 and PM3**
**Reichert et al.** 26	**Clinical trial**	**51 patients**	**Yes**	**37.95 ± 25.10% FOR PSG1 AND 33.96 ± 22.61% FOR PSG3**	**No**	**High agreement, high negative predictive value**
**Kingshott et al.** 28	**Clinical trial**	**16 patients**	**No**	**Yes: 382 ± 118**	**Yes: 37.95 ± 25.10% for PSG1**	**Better sleep quality on PM3, no difference in objective sleep on the next day**
**Masa et al.** 29	**Clinical trial**	**348 patients**	**Yes**	**442.5 ± 42.8 for PSG1 and 428.3 ± 81.9 for PM3**	**No**	**Lower AHI in PM3 by recording time significantly longer than sleep time**
**Ng et al.** 23	**Clinical trial**	**90 patients**	**Yes**	**402.7 ± 74.5 FOR PSG1 AND 477.3 ± 87.1 FOR PM3**	**Yes: 73 ** ± **0.25%**	**High sensitivity, specificity, and negative predictive value**
							**Hernández-Bendezú et al.** 30	**Cohort study**	**70 patients**	**No**	**Yes: 465 (276–546)**	**Yes: 76.3 (0.6–464.5)**	**PM3 with good signal quality even with patients moving around the city after PM3 has been connected**
							**Hsu et al.** 31	**Cohort study**	**215 hypertensive patients**	**No**	**No**	**56,6 ± 29,8%**	**The time spent in the supine position is a predictor of OSA.**
							**Oliveira et al.** 32	**Clinical trial**	**26 patients with COPD**	**Yes**	**No**	**No**	**High loss of PM3 signal suggests difficulty using nasal cannula**
**Guerrero et al.** 33	**Clinical trial**	**56 patients**	**Yes**	**Yes: 380.2 ± 66.0 for PSG1 and 401.9 ± 59.6 for PM3**	**Yes: 45.6 ± 24.2% for PSG1 and 46.6 ± 10.3% for PM3**	**PM3 together with detailed medical evaluation can be used in patients without high pretest probability, time in the supine position much shorter in PM3**
**Gjevre et al.** 34	**Clinical trial**	**47 patients suspected of OSA**	**Yes**	**No**	**33.96 ± 22.61% for PM3 and**	**No significant difference in time in the supine position**
**Vonk et al.** 35	**Cohort study**	**POSA and non-apneic snoring patients**	**No**	**No**	**43.1% during the PSG1 night phase compared with 28.6% for PSG3**	**Using the PSG1 apparatus leads to an increase in the percentage of supine sleeping position causing an overestimation of OSA severity**
**Levendowski et al.** 36	**Clinical trial**	**37 patients**	**Yes**	**No**	**No**	**Results showed less variation in the night-to-night AHI in PM3 than in PSG1**
**Ng et al.** 37	**Clinical trial**	**50 patients**	**Yes**	**Yes: 402.7 ** ± **74.5 for PSG1 and 477.3 ** ± **87.1 for PSG3**		**Significant test sensitivity and specificity**

**Abbreviations:** AHI, apnea-hypopnea index; COPD, chronic obstructive pulmonary disease; OSA, obstructive sleep apnea; PM3, level 3 portable monitor; POSA, obstructive sleep apnea; PSG1, type-1 polysomnography; PSG3, type-3 polysomnography.

## Discussion

### The Ideal Recording Time for PM3

In the present review, we identified only 1 article that directly sought to determine the minimum recording time in PM3. Wittine et al. 22 (2014) retrospectively evaluated 129 patients undergoing OCST. They divided the exam into time windows and compared these partial results with the total. Based on these analyses, the authors 22 suggest the ideal minimum time for recording is 300 minutes. It is important to note that this study did not compare the test investigated with PSG1 and did not investigate the influence of the time spent in the supine position on this outcome. 22 It is yet not clear how many hours of recording are necessary for pm3 to keep the high sensitivity, specificit and negative predictive value demonstrated in other studies. 23

Most studies reviewed randomly determined that three hours of recording time was necessary to validate the test. Cheliout-Heraut et al. 24 (2011) randomly chose five hours to include PM3 records as valid compared with PSG1. Additionally, Yin et al. 27 (2006) showed that data from patients with at least 390 minutes of PM3 recording time ( *p* = 0.028) agreed better with PSG1 data.

It has been recommended that individuals with insomnia as comorbidity should not be indicated for a PM3 OSA diagnosis. Planès et al. (2010) applied PSG1 and PM3 tests on the same night to 50 coronary artery disease patients and concluded that PM3 might underestimate the AHI, especially in patients with reduced sleep efficiency. These findings indicate the importance of the recording time in the final result on the AHI.

Driver et al. 25 (2011) also observed the effect of short total sleep time compared with recording time underestimating the AHI. The authors 25 excluded shorter exams (“split night”) from the analysis when comparing PSG1 and PM3, significantly increasing the agreement between the exams. However, they failed to define the ideal time interval. Indeed, they reached the conclusion that, when dealing with a low AHI in patients with a high degree of suspicion, a subjective inquiry about sleep time should be made. 25

### Comparison of the Recording Time (PSG1 versus PM3)

The lack of technical supervision does not seem to shorten the average recording time during the PM3 test. 26 On the contrary, longer recording times during the PM3 have been reported. 27 Interestingly, in PM3 home monitoring, sleep efficiency, rapid eye movement (REM) sleep, and slow-wave sleep increase, maybe because the test becomes more comfortable without EEG monitoring. Furthermore, fewer instances of sleep fragmentation have been observed in PM3. 28

Despite the average recording time being adequate in the studied groups, the percentage of repeated exams due to short recording times can reach 10% depending on the population evaluated. 29

### Signal Loss in PM3

Since the purpose of the PM3 is to simplify OSA diagnosis and reduce its costs, the rate of signal loss leading to the need to repeat the PM3 or request a PSG1 is exceptionally relevant. In the literature, the percentage of repeated tests due to poor signal quality ranges from 13% 29 to 5%. 23 27 Most patients, when well-oriented, maintained monitoring for a minimum time of 240 minutes with acceptable signal loss. 27 30 31 A study 32 published in 2012 reported that 26% of exams were disregarded due to signal loss in patients with chronic obstructive pulmonary disease (COPD). This loss of signal can occur for only part of the night. We speculate that if we can reliably determine the minimum recording time in the PM3, fewer exams may be repeated if there is signal loss in just a few fragments of the night.

### Conflicting Results Regarding Recording Time in Supine the Position and the AHIsup (PSG1 versus PM3)

To determine whether the position interferes with the results on the PM3, we first need to identify whether, in the PM3, the patient tends to assume the supine position less frequently. The results in the literature are not homogeneous. The type of equipment used may explain these divergences since some portable devices are positioned on the patient's chest, which can increase the time spent in the supine position.

Based on time in the supine position and the AHIsup, Guerrero et al. 33 (2014) found no significant difference when studying patients without high pretest probability or with comorbidities when comparing the PSG1 and the PM3 for three consecutive nights. Similarly, Gjevre et al. 34 (2011), submitting 47 patients to the PSG1 and PM3 with an interval of 1 week, did not identify a significant difference in terms of time in the supine position and the AHIsup.

On the other hand, Yin et al. found that patients spent significantly less time in the supine position in the PM3 compared with PSG1. To further analyze that observation, they divided their patients into two groups: one that spent more time in the supine position in the PM3 and another that spent less time. They 27 observed that the agreement of the AHI between the tests was significantly lower in the group that spent less time in the supine position in the PM3.

In a study by Mello et al. (2022), the participants who underwent the PSG1 spent more time in the supine position and less time in the prone position than those who underwent the PM. Additionally, patients with OSA spent more time in the supine position, regardless of the diagnostic device used. Time spent in the supine position was also independently predicted by study type, body mass index (BMI), gender, and OSA diagnosis. 17

Similarly, a large-scale retrospective study including POSA and non-apneic snoring patients compared body position during the PSG1 in one night and during another night without the use of the PSG1 apparatus. The results indicated that using the PSG1 apparatus increased the percentage of sleeping in the supine position, which caused an overestimation of obstructive sleep apnea (OSA) severity. 35

The PSG1 test, which involves more complex monitoring, seems to interfere with the patients' comfort, forcing them to assume a non-habitual position. Levendowski et al. 36 (2009) Performed two nights of PSG1 with an average interval of a few weeks, found a substantial variation time spend in supine position and in AHI. This result did not occur when performing two nights of PM3 testing, during which minor variations were observed regarding the time in the supine position and the AHI. The authors 36 concluded that the greater comfort felt by the patient during the exam minimized the “first-night” effect, which is the impact of an unusual set of monitors on the patients' habitual sleep.

In a study by Ng et al. 37 (2010), a few patients assumed a non-supine position while being monitored on the same night with PSG1 and PM3. The influence of the PSG1 in changing patients' habitual sleep position, thus changing the AHI, deserves further investigation.

## Conclusion

The high sensitivity, specificity, and negative predictive value of the PM3 have already been demonstrated in patients with high pretest OSA probability. The high variation in the rate of missed exams for excessively short recording time or signal loss in different studies reinforces the need for patient education strategies when considering this test. Furthermore, it is crucial to establish if the recording time was appropriate for diagnosis and therapeutic decision-making. Future studies are needed to elucidate fundamental issues such as the minimum recording time and if the time the patients spend in the supine position can affect the results.
